# *Wolbachia* transinfections in *Culex quinquefasciatus* generate cytoplasmic incompatibility

**DOI:** 10.1111/imb.12604

**Published:** 2019-07-03

**Authors:** T. H. Ant, C. Herd, F. Louis, A. B. Failloux, S. P. Sinkins

**Affiliations:** ^1^ MRC‐University of Glasgow Centre for Virus Research University of Glasgow Glasgow UK; ^2^ Biomedical and Life Sciences Lancaster University Lancaster UK; ^3^ Department of Virology, Arboviruses and Insect Vectors Institut Pasteur Paris France

**Keywords:** Culex quinquefasciatus, Wolbachia, cytoplasmic incompatibility, incompatible insect technique, population replacement, transinfection

## Abstract

*Culex quinquefasciatus* is an important mosquito vector of a number of viral and protozoan pathogens of humans and animals, and naturally carries the endosymbiont *Wolbachia pipientis*, strain *w*Pip. *Wolbachia* are used in two distinct vector control strategies: firstly, population suppression caused by mating incompatibilities between mass‐released transinfected males and wild females; and secondly, the spread of pathogen transmission‐blocking strains through populations. Using embryonic microinjection, two novel *Wolbachia* transinfections were generated in *C. quinquefasciatus* using strains native to the mosquito *Aedes albopictus*: a *w*AlbB single infection, and a *w*Pip plus *w*AlbA superinfection. The *w*AlbB infection showed full bidirectional cytoplasmic incompatibility (CI) with wild‐type *C. quinquefasciatus* in reciprocal crosses. The *w*Pip*w*AlbA superinfection showed complete unidirectional CI, and therefore population invasion potential. Whereas the *w*AlbB strain showed comparatively low overall densities, similar to the native *w*Pip, the *w*Pip*w*AlbA superinfection reached over 400‐fold higher densities in the salivary glands compared to the native *w*Pip, suggesting it may be a candidate for pathogen transmission blocking.

## Introduction

*Culex quinquefasciatus* (Say), the southern house mosquito, transmits a number of important human and animal pathogens, including arboviruses such as West Nile and Rift Valley fever, and the filarial nematode *Wuchereria bancrofti* (Sudomo *et al*., 2010). It is also significant from the perspective of wildlife conservation, as it transmits avian malaria (*Plasmodium relictum*) and avian pox virus on the Hawaiian Islands, where it has been incriminated in the decline of several endangered bird species (Van Riper *et al*., 1986). *C. quinquefasciatus* exhibits plasticity in host choice, frequently biting humans and other mammals as well as birds, and as such has the potential to act as a bridge vector for zoonotic pathogens (Farajollahi *et al*., 2011). As a cosmopolitan species, it has a wide distribution throughout the tropics and subtropics where it is frequently associated with urban areas. The larval stages thrive in domestic water bodies polluted with organic matter, such as pit latrines, blocked drainage ditches, and shallow wells. Vector control is generally limited to insecticide treatments and larval‐source management. Owing to predominantly night‐time biting and indoor resting, the distribution of insecticide‐treated nets and the use of indoor residual spraying for the control of malaria‐transmitting *Anopheles* species has applied concomitant selection on *C. quinquefasciatus* populations, with high levels of insecticide resistance reported in Africa (Norris and Norris, 2011; Jones *et al*., 2012; Yadouléton *et al*., 2015) and Asia (Yanola *et al*., 2015).

*C. quinquefasciatus* is a member of the *Culex pipiens* species complex, almost all populations of which are infected at close to 100% frequency with the maternally inherited intracellular endosymbiont *Wolbachia pipientis*, strain *w*Pip. *Wolbachia* is widespread throughout the phylum Arthropoda, where different strains induce a variety of reproductive manipulations to facilitate host population invasion. A common variant found in mosquitoes and other Diptera is a modification of the infected male germline that results in sterility unless a compensatory *Wolbachia*‐secreted rescue factor is present in the germline of infected females. This coupling of cytoplasmic incompatibility (CI) rescue with maternal transmission results in a relative reproductive advantage for *Wolbachia‐*infected females, providing a population invasion potential, with frequency thresholds for spread largely determined by the balance between the positive fitness effects of CI and negative effects on life‐history traits (Hancock *et al*., 2011; Hancock *et al*., 2016). In the *C. pipiens* species group, strain *w*Pip induces a particularly complex pattern of crossing types between populations, with both unidirectional and bidirectional CI observed at varying levels of penetrance (Barr, 1980; Magnin *et al*., 1987; O'Neill and Paterson, 1992; Guillemaud *et al*., 1997; Sinkins *et al*., 2005; Walker *et al*., 2009; Bonneau *et al*., 2018).

CI provides a mechanism of sterility that can be used to reduce the reproductive potential of a population through the mass‐release of males (Laven, 1967; Dobson *et al*., 2002; Zabalou *et al*., 2004; Atyame *et al*., 2011; Calvitti *et al*., 2012; Chen *et al*., 2013; Atyame *et al*., 2016); the development of highly efficient automated sex separation technology makes this feasible on a large scale (Gilbert and Melton, 2018). The natural incompatibilities between *w*Pip variants within the complex could in theory be utilized for sterile male releases; however, it would be highly desirable for practical purposes to select a single ‘universal’ line adapted to mass rearing that generates sterility with the females of all target populations. To do so, it will be necessary to create transinfections with *Wolbachia* strains introduced from other host species.

*Wolbachia* has also been shown to possess a strong pathogen‐blocking capacity when some novel *Wolbachia*–host combinations are generated (Moreira *et al*., 2009; Bian *et al*., 2010; Kambris *et al*., 2010; Walker *et al*., 2011; Blagrove *et al*., 2012; Ant *et al*., 2018). *Aedes aegypti* transinfected with the *w*AlbB *Wolbachia* strain, for example, show strong transmission blocking of a number of arboviruses (Bian *et al*., 2010; Ant *et al*., 2018), including dengue, whereas *w*AlbB‐transinfected *Anopheles stephensi* show reduced *Plasmodium falciparum* oocyst and sporozoite loads (Bian *et al*., 2013). Artificial germline transinfection with *Wolbachia* has so far been limited to *Ae. aegypti* (Xi *et al*., 2005; Moreira *et al*., 2009; Walker *et al*., 2011; Blagrove *et al*., 2012), *Aedes albopictus* (Blagrove *et al*., 2012; Ant and Sinkins, 2018) and *An. stephensi* (Bian *et al*., 2013). The extension of *Wolbachia* transinfection generation to *Culex* or other vector species, to allow the exploration of either transmission blocking for replacement strategies or the generation of sterile males for suppression, has been encumbered by the technical challenges inherent in generating stable infections in the laboratory. Here we report the generation of two novel transinfections in *C. quinquefasciatus* with *Wolbachia* strains native to *Ae. albopictus*, including a native‐plus‐novel strain superinfection. The relative densities achieved by the transinfections, CI crossing patterns, the effects of the novel strains on host fecundity and immune gene expression are presented.

## Results

### *Generation of* w*AlbB and* w*Pip*w*AlbA lines in* C. quinquefasciatus

A *Wolbachia*‐free *C. quinquefasciatus* line PelU was previously created by antibiotic treatment of a wild‐type *w*Pip‐carrying Sri Lankan PelA colony (Pinto *et al*., 2013). A *w*AlbB transinfection was generated by transferring cytoplasm from eggs of a *w*AlbB‐carrying *Ae. aegypti* line to PelU embryos. A total of 420 PelU embryos were microinjected with *w*AlbB (Table 1). The *w*AlbB‐carrying *C. quinquefasciatus* line was generated from a single G_0_ female. Females of the *w*AlbB line were outcrossed to PelU males for five generations before a stable inbreeding colony was established. Maternal transmission rates of *w*AlbB when PelU males were crossed to *w*AlbB females (ie in the absence of CI) were found to be 100% from 200 progeny assessed.

**Table 1:** Microinjection statistics for strain generation. ‘Total embryos injected’ is the number of *Culex quinquefasciatus* embryos microinjected with each *Wolbachia* strain for each of the *w*Pip and PelU lines. ‘Total adults emerged’ is the number of microinjected embryos surviving to produce adults, with parentheses showing percentage. ‘Total positive G_0_ females’ is the number of resulting adult female mosquitoes that were PCR positive for the transinfecting *Wolbachia* strain. ‘Total G_0_‐G_1_ maternal transmission’ shows numbers of G_0_ females that successfully produced progeny positive for the transinfecting *Wolbachia* strain, with parentheses showing percentage of females displaying transmission out of total positive G_0_ females

*Wolbachia* strain	*w*AlbB		*w*AlbA		*w*Mel	
Donor species	*Aedes aegypti*		*Ae. aegypti*		*Ae. aegypti*	
Recipient *C. quinquefasciatus* strain	*w*Pip	PelU	*w*Pip	PelU	*w*Pip	PelU
**Total embryos injected**	680	420	580	660	780	940
**Total adults emerged (%)**	111 (16)	78 (19)	58 (10)	36 (5)	102 (13)	107 (11)
**Total positive G**_**0**_ **females**	20	18	8	4	12	18
**Total G**_**0**_ **–G**_**1**_ **maternal transmission (%)**	0	2 (11)	2 (25)	0	0	0

A superinfected *C. quinquefasciatus* line carrying both *w*AlbA and *w*Pip was established through transfer of cytoplasm from the eggs of *w*AlbA‐transinfected *Ae. aegypti* to embryos of the PelA (wild‐type *w*Pip‐infected) colony. A total of 580 embryos were microinjected with *w*AlbA (Table 1). The *w*Pip*w*AlbA line was established from the progeny of a single superinfected G_0_ female. Females from this line were backcrossed for five generations to males of the *w*Pip line before a stable inbreeding colony was established. *w*Pip*w*AlbA females were crossed to PelU males to evaluate rates of maternal inheritance in the absence of CI. Strain‐specific PCR indicated that the superinfection was transmitted at 100% fidelity from 200 progeny assessed.

Attempts were also made to generate a line carrying *w*Mel, a *Wolbachia* strain native to the fruit fly *Drosophila melanogaster*. Embryos from a transinfected strain of *Ae. aegypti* were used as the source of *w*Mel, and although more than 1700 embryos of the *w*Pip and PelU lines were injected, far more than for *w*AlbA and *w*AlbB, no stable transinfection was generated (Table 1).

### *CI crossing patterns and fecundity*

**Figure 1:**
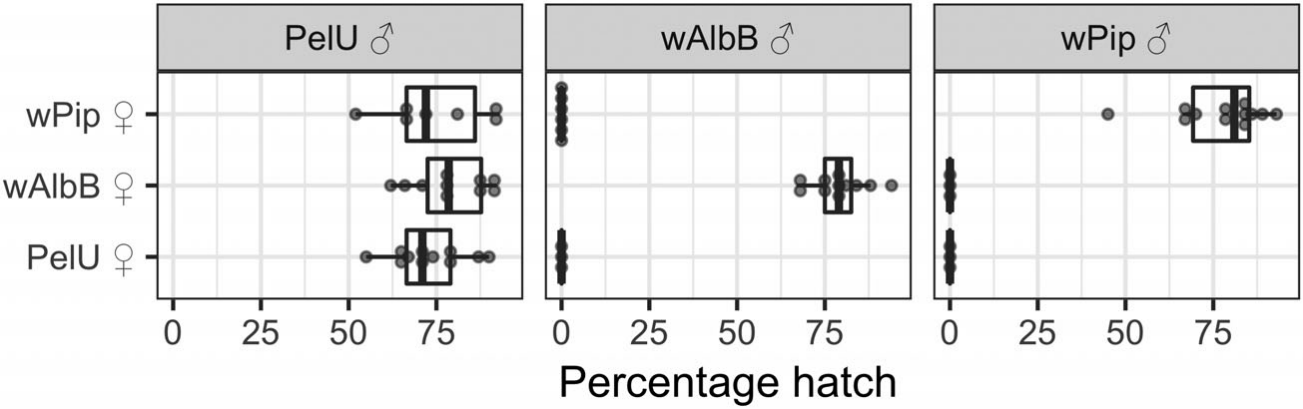
Percentage egg hatching rates from individual egg rafts resulting from crosses between the wild‐type *Wolbachia w*Pip, the *w*AlbB and the *Wolbachia* ‐ve (PelU, antibiotic‐treated) lines. Boxplots show median values and interquartile ranges. Dots show hatching rates from individual egg rafts.

Crosses were set up between the transinfected, *w*Pip (wild‐type) and PelU lines. No eggs hatched from reciprocal crosses between the *w*AlbB line and the *w*Pip line, displaying a classical pattern of complete bidirectional CI (Fig. 1). Egg hatch rates from crosses between PelU males and *w*AlbB females were not significantly different from wild‐type hatch rates (*p* = 0.077, Fisher's exact test), suggesting little effect of *w*AlbB on embryonic viability.

**Figure 2:**
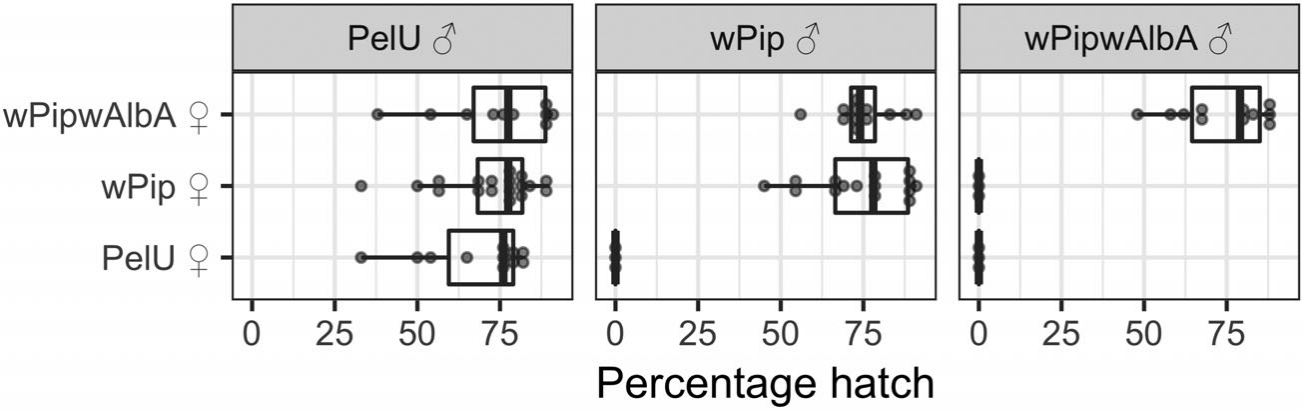
Percentage egg hatching rates from individual egg rafts resulting from crosses between the wild‐type *w*Pip, the *w*Pip*w*AlbA and the *Wolbachia*‐ve (PelU, antibiotic‐treated) lines. Boxplots show median values and interquartile ranges. Dots show hatching rates from individual egg rafts.

When males of the *w*Pip*w*AlbA line were crossed to *w*Pip females no egg hatching was observed, whereas *w*Pip*w*AlbA females were fully compatible with *w*Pip males and displayed no reduction in hatch rates compared to *w*Pip within‐strain crosses (*p* = 0.586, Fisher's exact test), suggesting full *w*Pip CI rescue (Fig. 2). The *w*Pip*w*AlbA line therefore displayed a classical pattern of complete unidirectional CI with wild‐type *C. quinquefasciatus*. Eggs resulting from crosses between females of the *w*Pip*w*AlbA line and *Wolbachia*‐free males showed similar hatch rates to those seen for the *w*Pip colony (*p* = 0.238, Fisher's exact test), suggesting little or no negative effects of the *Wolbachia* superinfection on embryo hatch rates in non‐CI crosses.

**Figure 3:**
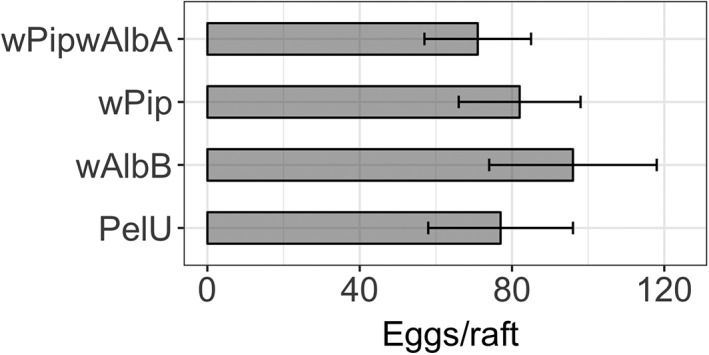
Average egg number per egg raft from *Wolbachia*‐transinfected, *w*Pip and PelU lines over the first gonotrophic cycle. Eggs from the rafts of 12–15 females were counted. Error bars show standard deviation.

The effects of *Wolbachia* infection status on the mean number of eggs produced by a female in an egg raft was assessed. No significant effect of *Wolbachia* infection status or strain was detected [Fig. 3; *p* > 0.4 for all comparisons, one‐way analysis of variance (anova) with Dunnett's], indicating that the presence of non‐native *Wolbachia* did not result in a reduction in fecundity, at least over the first gonotrophic cycle.

### Wolbachia *densities*

**Figure 4:**
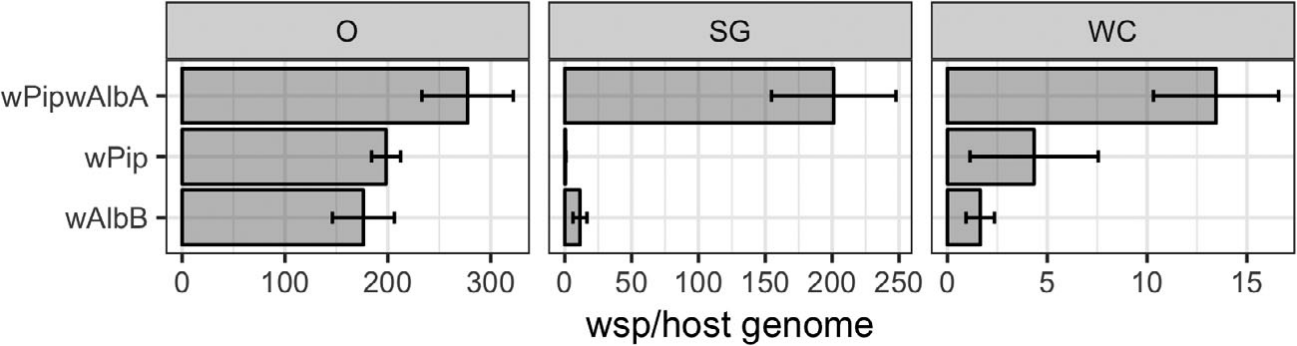
*Wolbachia* densities in ovary (O), salivary glands (SG) and whole female carcasses (WC) for the *w*Pip*w*AlbA, *w*AlbB and *w*Pip lines. Bar charts show mean densities and error bars show standard deviation. Each bar summarizes data from five biological repeats, each with either three whole female carcasses, or the dissected tissues from five females. wsp, *Wolbachia surface protein*.

Total *Wolbachia* densities were measured in 5‐day‐old whole female carcasses, dissected salivary glands and ovary tissue (Fig. 4). The *w*AlbB line displayed the lowest whole carcass density, with a mean of 1.64 (±1.11 SD) *Wolbachia* per host genome copies, significantly lower than the 4.34 (±1.68 SD) *Wolbachia* per host genome for the native *w*Pip strain (*p* = 0.014, one‐way anova with Dunnett's). The *w*Pip*w*AlbA superinfection reached a significantly higher density than wild‐type with a mean of 13.45 (±6.19 SD) *Wolbachia* per host genome copies (*p* = 0.00765, one‐way anova with Dunnett's). Densities of *Wolbachia* in the ovaries were not found to vary between the transinfected and wild‐type line (*p* > 0.075 for both comparisons, one‐way anova with Dunnett's). For the salivary glands, however, a significantly higher mean density was observed for the *w*Pip*w*AlbA superinfection compared to the *w*Pip strain alone (*p* < 0.0001, one‐way anova with Dunnett's), with 200.84 (±47.31 SD) compared to 0.494 (±0.36 SD) *Wolbachia* per host genome copies, respectively. The *w*AlbB strain showed a mean salivary‐gland density of 8.59 (±7.23 SD) *Wolbachia* per host genome, a nonsignificant difference compared to *w*Pip (*p* = 0.072, one‐way anova with Dunnett's).

### *Immune gene expression*

**Figure 5:**
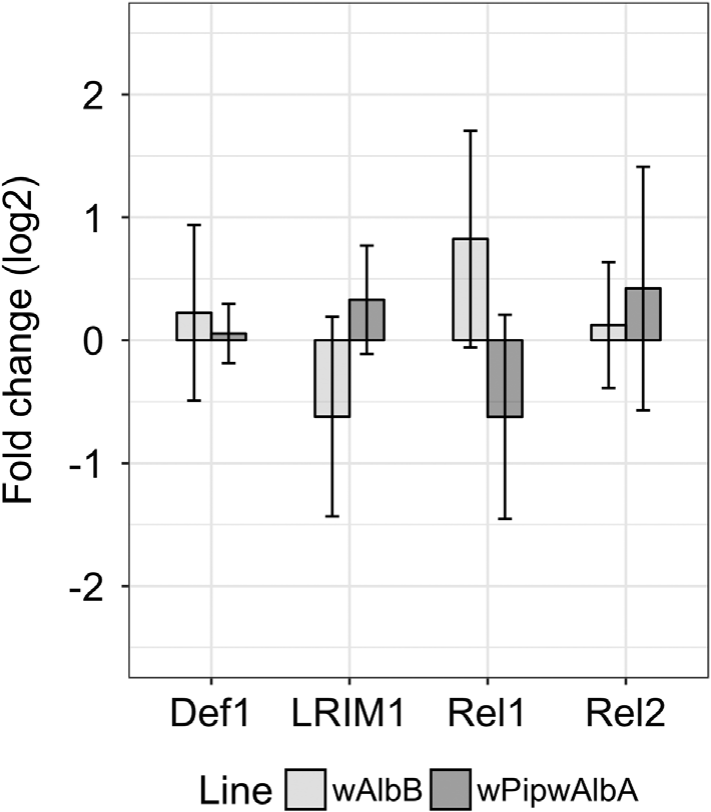
Expression of immune genes in the *w*AlbB and *w*Pip*w*AlbA lines normalized initially to the *18S ribosomal RNA* house‐keeping gene and then to expression in the *w*Pip line. Error bars show 95% confidence intervals from five biological replicates, each containing cDNA from a pool of three females. *Def1*, *Defensin1*; *LRIM1*, leucine‐rich repeat immune protein 1; *Rel1*, a homologue of *Drosophila dorsal*; *Rel2*, an NF‐κB transcriptional factor.

The transcription of a selection of immune genes was measured in whole adult females of the *w*AlbB and *w*Pip*w*AlbA lines and was compared to transcription levels in *w*Pip females. Immune genes investigated were: *Rel1* (a homologue of *Drosophila* dorsal) and *Rel2* (an *NF‐κB transcriptional factor*), regulators of the Toll and IMD pathways respectively, *Defensin1*, which can be activated through both Toll and Immune deficiency pathway (IMD) signalling, and the leucine‐rich repeat immune protein 1 (*LRIM1*), part of the complement‐like pathway. No significant effect of either *Wolbachia* strain was found on immune gene transcription (*p* > 0.2, one‐way anova with Dunnett's) (Fig. 5).

## Discussion

The two novel *Wolbachia* transinfections in *C. quinquefasciatus* reported here could potentially contribute to control in two ways: by providing a source of sterile males for population suppression, and through pathogen transmission blocking via population replacement. Males of the *w*AlbB‐only infection and the *w*Pip*w*AlbA superinfection both caused fully penetrant CI when crossed to wild‐type females. Females of the *w*AlbB line were also incompatible with wild‐type males, a bidirectional CI pattern resulting in high invasion thresholds, ideal for a suppression strain. No significant effect of *w*AlbB was observed on host fecundity. This suggests the line is relatively fit compared to wild‐types, an important factor given successful suppression would depend on the mass‐rearing and release of large numbers of fit, competitive incompatible males. Females of the *w*Pip*w*AlbA line were fully compatible with wild‐type males. The superinfection is thus expected to have the capacity to invade and establish in wild populations of the same crossing type as the Sri Lankan Pel wild‐type line. Although novel *Wolbachia* transinfections have been shown to decrease fecundity in some instances (Hoffmann *et al*., 2014), probably reducing strain invasiveness (Schmidt *et al*., 2017), no significant effects of the *w*Pip*w*AlbA superinfection were found on fecundity – although an impact of the infection on other life‐history traits such as longevity cannot be ruled out. *w*Pip*w*AlbA in *C. quinquefasciatus* provides a further example of additive CI, with modification and rescue of co‐infecting strains expressed independently (Dobson *et al*., 2004; Joubert *et al*., 2016). However, additive superinfection CI is not always stable; a *Wolbachia* triple infection in *Ae. albopictus* suggested co‐infecting strain interaction, affecting densities and CI rescue of co‐infecting strains (Ant and Sinkins, 2018). Attempts to generate a *w*Mel infection in *C. quinquefasciatus* were unsuccessful. The relatively high numbers of positive G_0_ females generated with no resulting G_0_–G_1_ transmission suggests that there may be factors limiting the transmissibility of *w*Mel in this species.

*Wolbachia* intracellular density correlates positively with levels of pathogen inhibition (Lu *et al*., 2012), although there is considerable between‐strain variability in blocking capacity (Martinez *et al*., 2014; Ant *et al*., 2018). Surprisingly, we found lower average densities for *w*AlbB compared to the native *w*Pip infection. This was unexpected as novel transinfections tend to show greater somatic tissue dispersal (McGraw *et al*., 2002), and thereby higher overall densities than native strains. As somatic infections can have deleterious effects on fitness, co‐evolutionary pressures acting on both host and symbiont are expected to favour mechanisms that restrict tissue tropism to the testes and ovaries given CI and transovarial transmission. These factors appear to be strain‐ and host‐specific; the native *Wolbachia* strains in female *Ae. albopictus* for example, particularly *w*AlbA, are largely localized to the ovaries and testes, whereas the non‐native *w*Mel can be found at high density in somatic tissues (Ant and Sinkins, 2018). A possible explanation for the low density of *w*AlbB in *C. quinquefasciatus* is the close phylogenetic relationship of *w*AlbB and *w*Pip (Ellegaard *et al*., 2013), with mechanisms selected to restrict *w*Pip in somatic tissues also functioning with *w*AlbB. As high densities also tend to result in reduced fitness (Chrostek *et al*., 2013; Sinkins, 2013; Fraser *et al*., 2017; Ant *et al*., 2018), the finding that *w*AlbB achieves low densities in *C. quinquefasciatus* suggests that any fitness costs in this line may be minimal, important for mass‐rearing and mate competition; however, it does also suggest that there will be limited pathogen inhibition potential.

The *w*Pip*w*AlbA transinfection was found to have an approximately threefold greater whole carcass density than the *w*Pip‐only native infection in the PelA line. This appears to be the result of a greater distribution of *Wolbachia* in somatic tissues, with a 400‐fold higher density observed in the salivary glands. A high *w*AlbA density is consistent with previous results from a transinfection in *Ae. aegypti*, where *w*AlbA was found to reach higher densities than a range of other strains, including *w*AlbB (Ant *et al*., 2018). This contrasts with the relative densities of the two strains in the native *Ae. albopictus*, where *w*AlbA reaches approximately 10% of the density of *w*AlbB (Dutton and Sinkins, 2004); again, co‐evolutionary pressures have probably selected for reproductive tissue localization in the native host. Experiments carried out in *Ae. aegypti* showed a low virus inhibition potential for *w*AlbA against the model arbovirus Semliki Forest virus (Ant *et al*., 2018) following intrathoracic viral challenges, but it is nevertheless able to block transmission of Zika using oral challenges (Chouin‐Carneiro *et al*., 2019). West Nile and Zika are related flaviviruses, and thus *w*AlbA may have transmission‐blocking potential in *Culex*.

*C. quinquefasciatus* is a competent vector for a wide variety of pathogens, ranging from viruses including West Nile and Rift Valley fever, to eukaryotes including the protozoan *P. relictum* and the filarial nematode *Wu. bancrofti*. Experimental results from a range of host species suggest that the mechanism of *Wolbachia*‐mediated pathogen inhibition differs between viruses and eukaryotic parasites. *Plasmodium* and filarial inhibition probably depends at least in part on a priming of the host innate immune system (Kambris *et al*., 2009; Kambris *et al*., 2010; Bian *et al*., 2013). *Wolbachia* transinfections in *Ae. aegypti* activate a range of immune signalling pathways, including the Toll, Imd and complement‐like pathways (Kambris *et al*., 2009; Moreira *et al*., 2009; Rancès *et al*., 2012). *An. gambiae* somatically infected with *w*MelPop block *Plasmodium berghei* development, which can be restored by knock‐down of the Thioester containing protein 1 (*TEP1*) opsonin (Kambris *et al*., 2010). No immune priming was detected in the transinfections of *C. quinquefasciatus* presented here, which included examining defensin, an antimicrobial peptide that was very highly upregulated in *w*MelPop‐, *w*Mel‐ and *w*AlbB‐infected *Ae. aegypti* (Bian *et al*., 2010; Rancès *et al*., 2012). This lack of immune upregulation suggests that any blocking of eukaryotic parasites in these *Wolbachia* transinfections may be limited. In contrast, *Wolbachia*‐mediated blocking of viruses does not appear to require immune priming (Blagrove *et al*., 2012; Rancès *et al*., 2012, 2013; Molloy and Sinkins, 2015). Evidence from *Ae. aegypti* cells infected with *w*MelPop and challenged with dengue suggest that blocking is the result of disruption of host cell lipid homeostasis and accumulation of cholesterol in lipid droplets (Geoghegan *et al*., 2017). A previous study investigating the immune priming of a transinfection of *w*Mel in *Ae. albopictus* also found very low levels of immune gene upregulation (Blagrove *et al*., 2012; Molloy and Sinkins, 2015), suggesting that the immune response of natively infected species may have an innate desensitization to the presence of *Wolbachia*. The demonstration of strong dengue and chikungunya blocking by the high density *w*Mel infection in *Ae. albopictus* in the absence of immune priming is encouraging for the potential for viral inhibition in the *w*Pip*w*AlbA *C. quinquefasciatus* line presented here.

## Experimental procedures

### *Lines and rearing*

The *C. quinquefasciatus* wild‐type was the Pel line originally colonized in Sri Lanka. The *Wolbachia*‐free PelU line was created by antibiotic treatment (Pinto *et al*., 2013). The source of *w*AlbA and *w*AlbB *Wolbachia* for cytoplasmic transfers was from transinfected *Ae. aegypti* colonies (Ant *et al*., 2018). All mosquito colonies were maintained at 27 °C and 70% relative humidity with a 12‐h light/dark cycle. Larvae were fed tropical fish pellets (Tetramin, Tetra, Melle, Germany) and adults were given access to a sucrose meal *ad libitum*. Bloodmeals were provided using a Hemotek artificial blood‐feeding system (Hemotek, Blackburn, UK) using defribrinated sheep blood (TCS Biosciences, Botolph Claydon, UK). Eggs were collected by providing a bowl of water for oviposition 3–4 days post blood‐feeding.

### *Transinfection generation*

The *w*AlbB *C. quinquefasciatus* line was generated by transferring cytoplasm from *w*AlbB‐infected *Ae. aegypti* into embryos derived from the PelU colony. The *w*Pip*w*AlbA superinfection was generated by transferring cytoplasm from *w*AlbA‐infected *Ae. aegypti* into embryos derived from the wild‐type PelA colony. Microinjections were performed using methods described previously (Blagrove *et al*., 2012) adapted for *Culex* mosquitoes. Briefly, ~30‐min‐old egg rafts were collected and individual eggs lined against a damp nitrocellulose membrane fixed to a glass microscope slide. Eggs were briefly dried (~1 min) and covered in Voltalef 10s oil (VWR International, Radnor, PA, USA) for injection. Injected eggs were monitored for 24 h, and neonate larvae removed from oil using a fine paint brush and placed in a bowl of water for development. Female G_0_ survivors were back‐crossed to wild‐type males, blood‐fed and separated individually for oviposition. G_0_ females were analysed for *Wolbachia* infection by strain‐specific PCR and eggs from *Wolbachia‐*negative G_0_ females were discarded. Eggs of *Wolbachia*‐positive females were hatched and G_1_s were assessed for *Wolbachia* G_0_–G_1_ germline transmission. In generating both the *w*AlbB and *w*Pip*w*AlbA lines, two separate G_0_ females with G_1_ transinfection transmission were derived. As duplicate transinfections carried the same *Wolbachia* strains in the same host background, only one line of each was carried forward for characterization – in both instances the G_3_ colony with the greatest number of individuals was chosen. Individual *Wolbachia* strains were screened using strain‐specific primers: 183F + 691R for *w*Pip; *w*AlbAF + *w*AlbAR for *w*AlbA; *w*AlbBF + *w*AlbBR for *w*AlbB. For sequences see Table 2.

**Table 2:** List of primer sequences used in this study

Primer name	5′–3′ sequence
Rel1‐F	GCGACTTTGGCATCAAGCTC
Rel1‐R	GTTCGACCGGAGCGTAGTAG
Rel2‐F	GTCGAGATGGCCAAAACGATG
Rel2‐R	TCATATTGTTGATGGCATT
LRIM1‐F	CGTAATGGTGCCAAGAGACA
LRIM1‐R	GGCGTAAGGTGCTGATGATT
Def1‐F	GGTCCAATACTTCGCCAATAC
Def1‐R	GATTGGGCGTCAACGATAGT
qWSP‐F	ATCTTTTATAGCTGGTGGTGGT
qWSP‐R	AAAGTCCCTCAACATCAACCC
qHTH‐F	TGGTCCTATATTGGCGAGCTA
qHTH‐R	TCGTTTTTGCAAGAAGGTCA
18S rRNA‐F	CGCGGTAATTCCAGCTCCACTA
18S rRNA‐R	GCATCAAGCGCCACCATATAGG
183F (Zhou *et al*., 1998)	AAGGAACCGAAGTTCATG
691R (Zhou *et al*., 1998)	AAAAATTAAACGCTACTCCA
*w*AlbB‐F	GCAATACCTATGCCGTTTA
*w*AlbB‐R	GACGAAGGGGATAGGTTAATATC
*w*AlbA‐F	GTAGTATTTACCCCAGCAG
*w*AlbA‐R	ATCTGCACCAGTAGTTTCG

Rel1, a homologue of Drosophila dorsal; Rel2, an NF‐κB transcriptional factor; LRIM1, leucine‐rich repeat immune protein 1; Def1, Defensin1; WSP, *Wolbachia* surface protein; HTH, homothorax; rRNA, ribosomal RNA.

### *Maternal inheritance, CI crosses, and fecundity*

To assess rates of maternal inheritance, females from the *Wolbachia* transinfected lines were crossed to PelU males in pools of 30 males and 15 transinfected females. A bloodmeal was provided and egg rafts collected and hatched individually. DNA from a selection of 10 larvae resulting from each egg raft (100 larvae assessed for each line in total) was extracted at the pupal stage and a PCR for *Wolbachia* was performed.

Rates of CI induction and rescue both with wild‐type mosquitoes and between infected lines were assessed by crossing 30 males and 15 females of each line. A bloodmeal was provided and egg rafts collected and hatched individually. Eggs were counted to assess female fecundity. Resulting larvae were counted at the L2‐L3 stage to provide hatching rates. Females with no eggs that hatched were dissected to check spermathecae for successful mating. Unmated females were excluded from hatch rate evaluations.

### *Density assessment*

For quantitative PCR analysis, genomic DNA was extracted from mosquitoes using phenol/chloroform. Mosquitoes used in density experiments were adults 5 days post pupal eclosion. Genomic DNA was diluted to 100 ng/μl using a NanoDrop spectrophotometer (Thermo Scientific, Waltham, MA, USA). A Bio‐Rad CFX‐96 real‐time PCR detection system was used (Bio‐Rad, Hercules, CA, USA) with 2 x SYBR‐Green mastermix (Biotool, Houston, TX, USA). Total *Wolbachia* density was analysed by relative quantification of the *Wolbachia surface protein* against the mosquito *homothorax* gene.

### *Immune gene expression*

Adult female RNA was extracted from four to five adult mosquitoes using TRIzol Reagent (Life Technologies, Carlsbad, CA, USA) following the manufacturer's instructions. TRIzol‐extracted RNA was DNase I treated and purified via standard phenol/chloroform extraction. cDNA synthesis was performed in a total reaction volume of 10 μl, using an iScript cDNA synthesis kit (Bio‐Rad). A Bio‐Rad CFX‐96 real‐time PCR detection system was used (Bio‐Rad) with 2 x SYBR‐Green mastermix (Biotool). Primers Def1‐F + Def1‐R, Rel1‐F + Rel1‐R, Rel2‐F + Rel2‐R and LRIM1‐F + LRIM1‐R were used to assess levels of *defensin 1*, *Rel1*, *Rel2* and *LRIM1*, respectively. Levels of target RNA sequences were normalized to the *18S ribosomal RNA* house‐keeping gene using the Pfaffl method. Primer sequences can be found in Table 2.
